# Finite Element Simulation of a Crack Growth in the Presence of a Hole in the Vicinity of the Crack Trajectory

**DOI:** 10.3390/ma15010363

**Published:** 2022-01-04

**Authors:** Abdulnaser M. Alshoaibi, Yahya Ali Fageehi

**Affiliations:** Mechanical Engineering Department, College of Engineering, Jazan University, Jazan 45142, Saudi Arabia; yfageehi@jazanu.edu.sa

**Keywords:** finite element method, linear elastic fracture mechanics, stress intensity factors, holes, ANSYS Mechanical R19.2, SMART crack growth

## Abstract

The aim of this paper was to present a numerical simulation of a crack growth path and associated stress intensity factors (SIFs) for linear elastic material. The influence of the holes’ position and pre-crack locations in the crack growth direction were investigated. For this purpose, ANSYS Mechanical R19.2 was introduced with the use of a new feature known as Separating Morphing and Adaptive Remeshing Technology (SMART) dependent on the Unstructured Mesh Method (UMM), which can reduce the meshing time from up to several days to a few minutes, eliminating long preprocessing sessions. The presence of a hole near a propagating crack causes a deviation in the crack path. If the hole is close enough to the crack path, the crack may stop at the edge of the hole, resulting in crack arrest. The present study was carried out for two geometries, namely a cracked plate with four holes and a plate with a circular hole, and an edge crack with different pre-crack locations. Under linear elastic fracture mechanics (LEFM), the maximum circumferential stress criterion is applied as a direction criterion. Depending on the position of the hole, the results reveal that the crack propagates in the direction of the hole due to the uneven stresses at the crack tip, which are consequences of the hole’s influence. The results of this modeling are validated in terms of crack growth trajectories and SIFs by several crack growth studies reported in the literature that show trustworthy results.

## 1. Introduction

Studying and evaluating mechanical and structural components’ integrity is a particularly important task in engineering. Internal crack growth is closely related to the quality and stability of engineering structures, according to a great amount of engineering practice. As a result, crack propagation path prediction and crack stability analysis are essential for predicting the integrity and durability of engineering structures. Many researchers have focused on using analytical or computational formulations to investigate dynamic fracture mechanics. Numerous numerical approaches for simulating crack propagation have been used, including the finite element method (FEM) [1], Discrete Element Method (DEM) [2,3,4], Element Free Galerkin method (EFGM) [5], extended finite element method (XFEM) [6,7], Cohesive Element Method (CEM) [8,9], Boundary Element Method (BEM) [10], meshless method [11,12] and Phase-Field Method (PFM) [13]. Most fracture mechanics models in the literature are developed within the framework of the finite element method (FEM), as this method is robust, reliable and deals with complex geometries [14,15,16,17,18,19,20,21,22]. Numerous fatigue crack models have been developed during the last 30 years based on numerical simulations to predict the fatigue life of practical engineering structures under service circumstances. Many three-dimensional software tools, including FRANC3D [23], ZENCRACK [24,25], ABAQUS [26,27], and BEASY [28], use these approaches. Damage tolerance analysis, based on crack tip SIFs, has become one of the most widely used applications of LEFM [29]. The main LEFM characteristics associated with crack propagation are SIFs, energy release rate, and J-integral. In the linear formulation, the last two factors are equivalent and pertain to the SIFs, whereas the crack growth and the evaluation of the front shape are predicted depending on the values of SIFs. The SIFs are used to determine the severity of the stress caused by remote loading in the vicinity of the crack tip. During crack growth, the related instantaneous value of SIFs would follow the changes in the crack geometry and stresses. The load, boundary conditions, crack propagation, geometry, and material properties all influence the stress intensity factor. For decades, FEM has been effectively used to analyze cracked structures. However, since a new mesh must be generated in the vicinity of a crack tip after every step of crack growth, the crack growth simulation is computationally very intensive. The modeling procedures have been significantly simplified and the computation time drastically reduced by the development of software such as the ANSYS workbench’s smart crack growth module. In this study, FEM was used with Ansys Workbench software, and crack propagation was simulated using genuine SMART, which only allows mesh adaptation in the vicinity of the crack. Crack propagation modeling is an incremental procedure in which a set of stages is repeated for each model progression. Each simulation increment is based on previously computed results and represents one crack configuration. The main motivation for this study was to make a significant contribution to the use of ANSYS as an efficient tool for modeling crack growth under mixed-mode loading conditions and monitoring the effect of the holes and the crack position on the crack growth trajectory.

## 2. ANSYS SMART Crack Growth

ANSYS is a powerful software program for finite element analysis. Ansys Parametric Design Language is used in this work to calculate SIFs, crack growth direction, and the stresses and strain distribution for two geometries, namely a cracked plate with four holes and a plate with a circular hole, and an edge crack with different pre-crack locations. There are several experimental models for crack growth modeling that have been presented [30,31,32]; however, they are both costly and time-consuming. Incorporating a simulation technique that includes numerical analysis and the usage of the ANSYS Mechanical R19.2 finite element method is an excellent way to save laboratory effort, time, and costs. Mesh generation is a critical step that influences the performance of finite element analysis. ANSYS Workbench provides a reliable and robust structural mesh generator capable of generating a consistent mesh for the entire structure with minimal computational effort. The element size was optimized using a mesh sensitivity analysis, which improved the precision of the results. The crack growth simulation and meshing processes in the ANSYS workbench are automated as well as an accurate prediction of the SIFs. There are three types of cracks that ANSYS can model: arbitrary, semi-elliptical, and pre-meshed. The unstructured mesh method (UMM) is used in the Separating, Morphing, Adaptive and Remeshing Technology (SMART) algorithm. At each solution step, SMART automatically updates the mesh to reflect changes in crack geometry caused by crack growth. Instead of using the enrichment technique, it uses a localized remesh function as the crack grows. Unlike the XFEM, SMART can be scaled up for larger projects since remeshing is limited to a small area around the crack tip at each iteration. After each iteration, remeshing around the crack tip concentrates computing power where it is most needed, making SMART simulations faster and easier to scale up for larger projects. This approach does not necessitate the development of new elements, and conventional elements included in ANSYS Mechanical can still be employed. The pre-meshed crack approach necessitates the use of a crack front as identified by the Smart Crack growth analysis tool. On each solution stage, SMART automatically updates the mesh from crack geometry changes due to crack propagation, decreasing the requirements for long pre-processing sessions. The crack tip mesh and the geometric edge that passes through the thickness were refined using the sphere of influence. The crack tip, the crack’s top surface, and the crack’s bottom surface are all included in the geometric regions. Each of these regions is connected to a set of nodes that will be utilized for the simulation.

For the static crack growth simulation, ANSYS Mechanical provides two common fracture criteria, which are the J-integral and stress intensity factor. In this study, the stress intensity factor criterion was used, which states that the crack grows when the equivalent stress intensity factor exceeds the fracture toughness of the material. This computation is performed along the distributed crack front, where the distribution of the stress intensity factor controls the adapting crack front shape. The equivalent stress intensity factor is represented by [33], as follows:

(1) $$\Delta K_{eq}=\frac{1}{2}\cos\frac{\theta }{2}\left[(\Delta K_{I}(1+\cos\theta ))-3\Delta K_{II}\sin\theta \right]$$

where: $\Delta K_{I}$ = the range of SIF in mode *I* loading and $\Delta K_{II}$ = the range of SIF in mode *II* loading.

The crack growth direction is identified by an angle determined from the original crack plane [34,35,36]. The ratio of stress intensity factor modes at the crack tip determines the direction of a mixed-mode crack. The maximum circumferential stress criterion is implemented in the ANSYS software for mixed-mode loading. The formula for the crack propagation direction in ANSYS is expressed as follows [33,37]:

(2) $$\theta =\cos^{-1}\;\left(\frac{3K_{II}^{2}+K_{I}\sqrt{K_{I}^{2}+8K_{II}^{2}}}{K_{I}^{2}+9K_{II}^{2}}\right)$$

where: *K_I_* = first mode of SIF and *K_II_* = second mode of SIF.

## 3. Results and Discussion

### 3.1. A Cracked Plate with Four Holes

**Figure 1:**
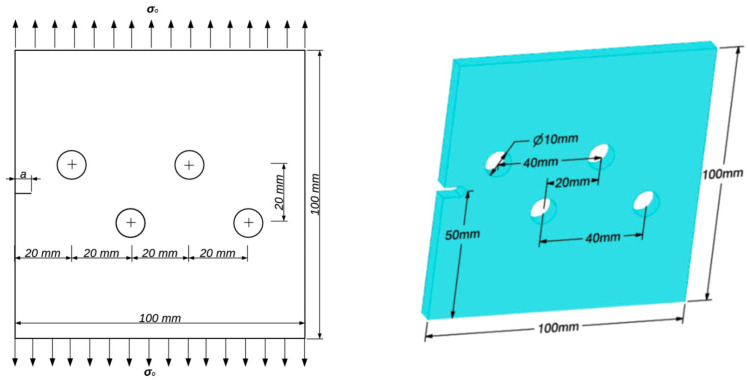
Cracked plate with four holes and one edge crack.

In this example, the crack growth path is deviated due to multiple holes in a relatively complicated shape. A square plate containing four holes of equal radii of 5 mm and a 6 mm long edge crack is considered, as shown in Figure 1. The plate is 100 mm × 100 mm × 10 mm in size. The dimensions of the geometry are 100 mm × 100 mm with two different thicknesses: 10 mm and 20 mm. Along the top and bottom edges of the plate, a uniform distributed stress of *σ* = 10 MPa is applied. The material is Aluminium 7075-T6 with the following properties: Modulus of elasticity, *E* = 72 GPa, Poisson’s ratio, *υ* = 0.33, Yield strength, *σ_y_* = 469 MPa, Ultimate strength, *σ_u_* = 538 MPa, Fracture toughness, *K_IC_* = 29 MPa $\sqrt{mm}$.

**Figure 2:**
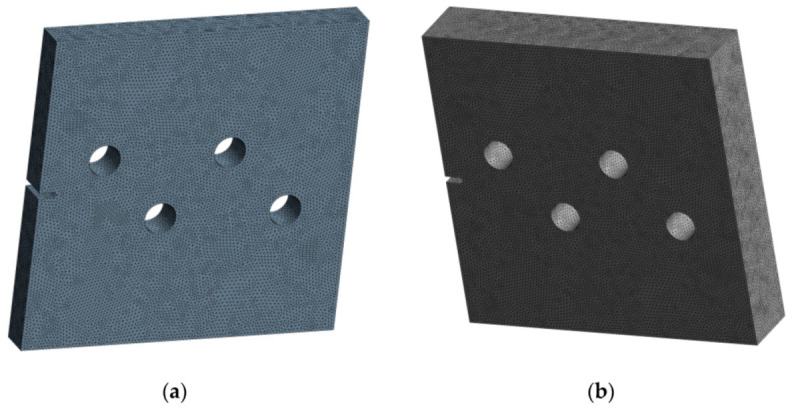
Initial mesh: (**a**) 10 mm thickness, (**b**) 20 mm thickness.

Figure 2 depicts the initial mesh generated by Ansys, which had a 1 mm element size and generated 693,495 nodes and 453,855 elements for a 10 mm (Figure 2a) geometry thickness and 762,845 nodes and 498,915 elements for a 20 mm geometry thickness (Figure 2b).

**Figure 3:**
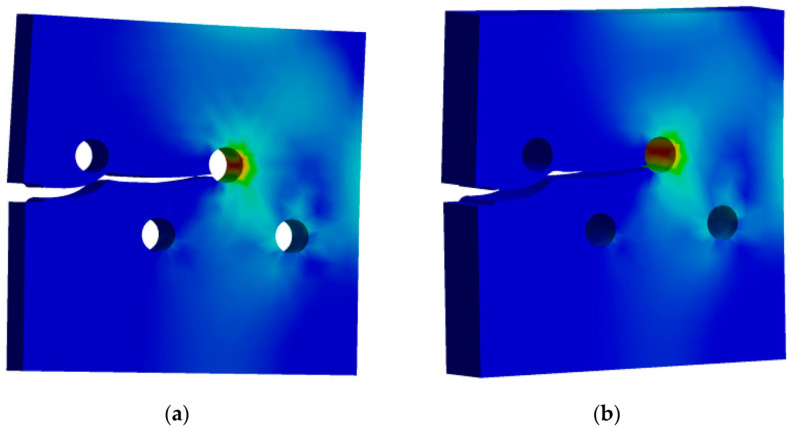
Predicted crack growth path: (**a**) 10 mm thickness, (**b**) 20 mm thickness.

Figure 3 shows the predicted crack growth path for two different thicknesses, Figure 3a for 10 mm thickness and Figure 3b for 20 mm thickness. As seen in Figure 3, the crack begins to grow in a straight line because there is no influence of holes. As it approaches the hole, it grows in the direction of the first upper hole, which was not close enough to cause the crack to sink into the hole; therefore, the crack has changed its direction and continues to propagate in a straight line until it arrives near the second upper hole, which is close enough to the crack trajectory for the crack to sink into it.

**Figure 4:**
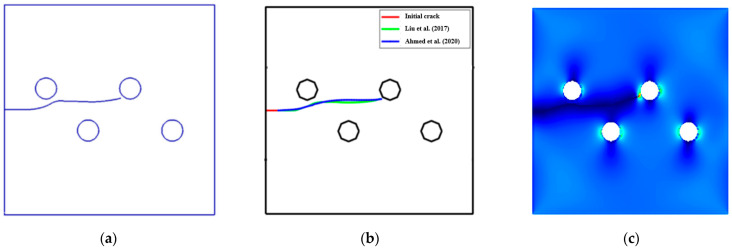
Crack growth trajectory: (**a**) Liu et al. FMM BEM [38], (**b**) Ahmed et al. [39], and (**c**) Wiragunarsa et al. [40].

As shown in Figure 4, the same problem was recently studied in two dimensions by Liu et al. [38] using the Fast Multipole Method (FMM) with the Boundary Element Method (BEM) (Figure 4a), by Ahmed et al. [39] using the boundary cracklet method (Figure 4b), and by Wiragunarsa et al. [40] using improved smoothed particle hydrodynamics (Figure 4c). A comparison of the crack propagation path using ANSYS and the reference study [38,39] is shown in Figure 4a,b, in which the crack growth direction shows a good agreement with the references. On the other hand, there was a slight deviation of the crack growth path predicted by Wiragunarsa et al. [40] (Figure 4c), when compared to the present study path as well as the other two referenced crack growth trajectories [38,39]. According to the authors’ [40] explanation, this deviation occurred due to a little numerical oscillation near the load located in the first step of applying the load.

**Figure 5:**
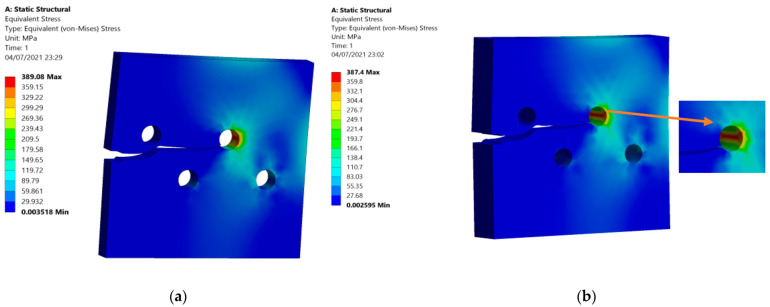
Von Mises stress distribution: (**a**) 10 mm thickness, (**b**) 20 mm thickness.

Figure 5a shows the Von Mises stress distribution for 10 mm thickness as well as, Figure 5b shows the Von Mises stress distribution for 20 mm thickness. As can be shown in this figure, there were no significant changes in the Von Mises stress due to the change in thickness, as the plane stress condition was assumed.

**Figure 6:**
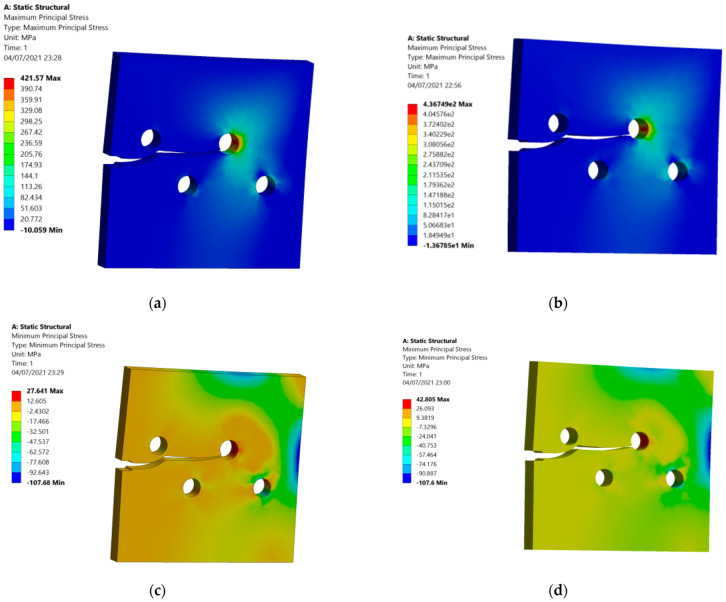
Crack growth trajectory: (**a**) maximum principal stress (10 mm thickness), (**b**) maximum principal stress (20 mm thickness), (**c**) minimum principal stress (10 mm thickness), (**d**) minimum principal stress (20 mm thickness).

The maximum and minimum principal stresses were compared for the two thicknesses side by side, as displayed in Figure 6. Figure 6a,b show the maximum principal stress distribution for the two different thicknesses, as well as Figure 6c,d show the minimum principal stress for both thicknesses.

**Figure 7:**
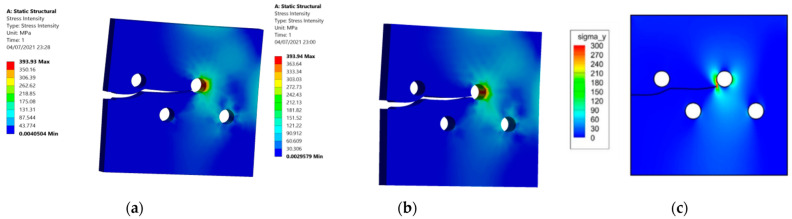
Stress intensity distribution: (**a**) 10 mm thickness, (**b**) 20 mm thickness, and (**c**) FMM BEM [38].

Figure 7 shows a comparison, with comparable results, of the stress intensity distribution between the present study results (Figure 7a,b) and those obtained by [38] using the Fast Multipole Method (FMM) with the Boundary Element Method (BEM) (Figure 7c).

**Figure 8:**
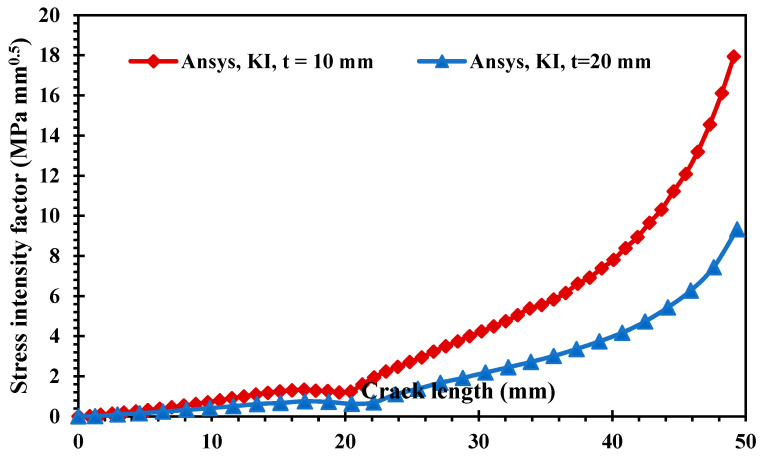
Results of the first mode of the stress intensity factor for different thicknesses.

**Figure 9:**
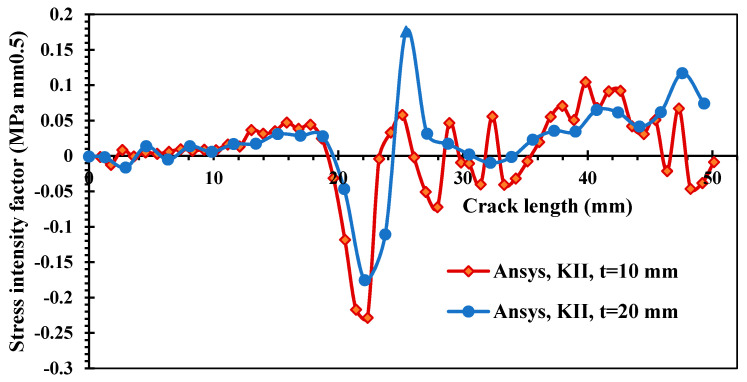
Results of the second mode of the stress intensity factor for different thicknesses.

Figure 8 and Figure 9 show the estimated results of the mixed-mode SIFs *K_I_* and *K_II_*, respectively, for the two different thickness values. The crack growth trajectory was accurately represented by these estimated values. As a result of the influence of the first upper hole, the values of *K_I_* were decreased in the crack length interval between 15 mm and 20 mm. On the other hand, the second mode of the stress intensity factor increased slightly in the same period, as shown in Figure 9. Subsequently, the value of KI started to increase again as the crack path missed the first upper hole until the crack reached the second upper hole. Figure 8 clearly shows the effect of different thicknesses on the first mode of SIFs. As the thickness increased, KI dropped by more than half after it passed the first upper hole.

### 3.2. A Plate with a Circular Hole and an Edge Crack with Different Pre-Crack Locations

**Figure 10:**
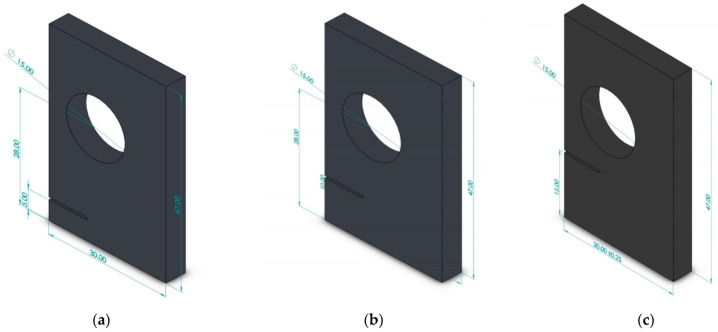
Dimensions of a plate with a hole and an edge crack with three different pre-crack locations, (**a**) h = 5 mm, (**b**) h =10 mm, and (**c**) h = 15 mm.

**Figure 11:**
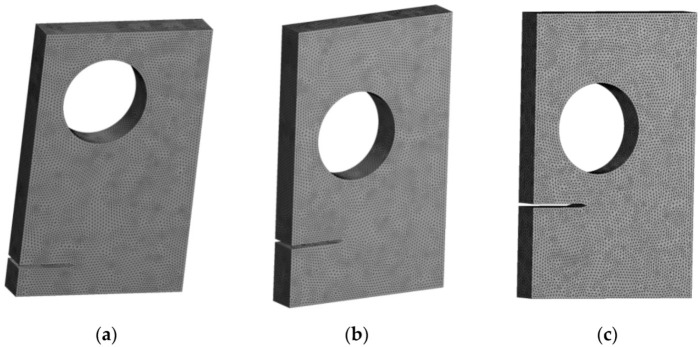
Initial mesh for the three configurations, (**a**) h = 5 mm, (**b**) h = 10 mm, and (**c**) h = 15 mm.

A single edge-cracked plate with a circular hole and multiple crack-tip locations was explored. Assuming a plane stress situation, three different pre-crack positions are investigated: h = 15 mm, h = 10 mm, and h = 5 mm. Figure 10 depicts the geometrical dimensions of these plates with three configurations. (Figure 10a with h = 5 mm, Figure 10b with h = 10 mm and Figure 10c with h = 15 mm). Figure 11 shows the initial mesh for the three configurations with an element size of 1 mm, with one round of mesh refinement generating a total number of nodes with 209,357 and 142,553 elements. The applied stress on the upper and lower edges is *σ* = 20 MPa, and the material has a Young’s modulus *E* = 1 GPa and a Poisson’s ratio *υ* = 0.3. (Figure 11a with h = 5 mm, Figure 11b with h = 10 mm and Figure 11c with h = 15 mm).

**Figure 12:**
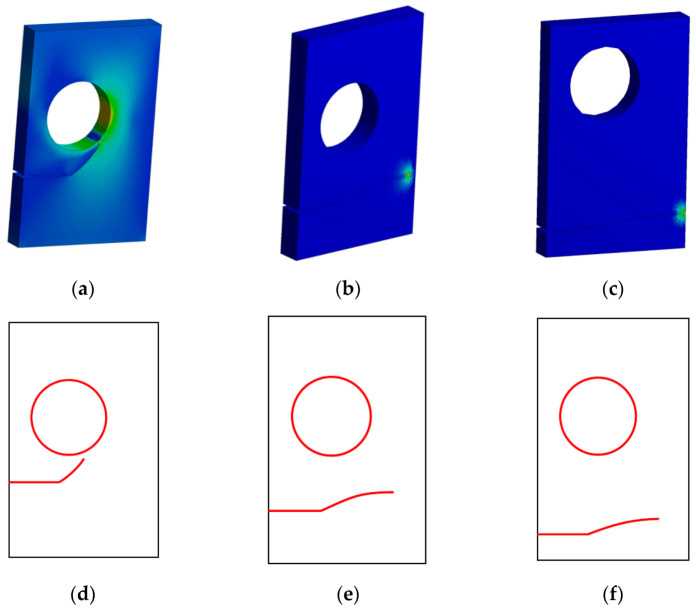
Crack growth trajectories, (**a**) h = 15, (**b**) h = 10, (**c**) h = 5, (**d**) h = 15 [41], (**e**) h = 10 [41], and (**f**) h = 5 [41].

Figure 12a–c shows the estimated crack propagation paths in the present study for three alternative crack position configurations, which were in agreement with the numerical results provided by Fang et al. [41] using a locally refined (LR) B-splines extended iso-geometric analysis (XIGA), as shown in Figure 12d–f. The crack trajectory is significantly influenced by the position of the pre-crack. The crack propagation analysis revealed that the crack initially always grew in the direction of the hole. At h = 5 mm and h = 10 mm, the crack growth path differed significantly from its initial direction and grew almost horizontally to the right side of the plate. Meanwhile, for h = 15 mm, as the location of the initial crack was closer to the hole, the deviation of the crack in the direction of the hole increased as it approached the hole until it sank into the hole. As a result, the closer the hole was to the crack, the more it influenced the crack’s propagation path.

**Figure 13:**
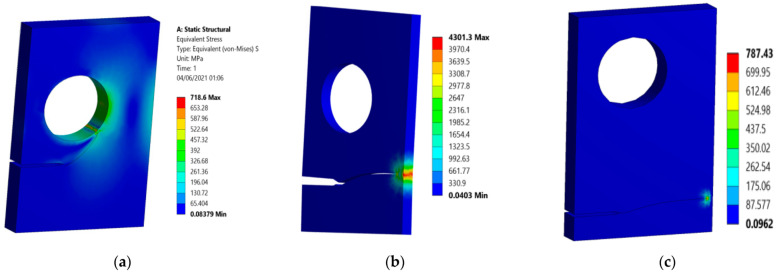

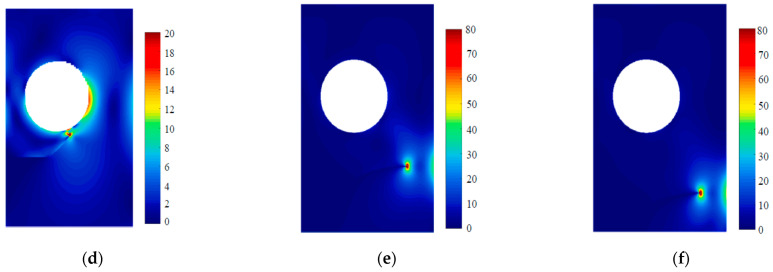
Von Mises stress distribution, (**a**) h = 15, (**b**) h = 10, (**c**) h = 5, (**d**) h = 15 [41], (**e**) h = 10 [41], and (**f**) h = 5 [41].

Figure 13 compares the Von Mises stress distributions in the present study (Figure 13a–c) with the numerically estimated values of Fang et al. [41] (Figure 13d–f). It is obvious that at h = 5 mm and h = 10 mm the stress was concentrated mainly around the crack tip, while at h = 15 mm the higher stress was mostly concentrated near the right side of the hole and around the crack tip. As a result, the crack was always attracted to the hole. It either curved and grew towards the hole or was deviated by the influence of the hole and grew out after missing it.

**Figure 14:**
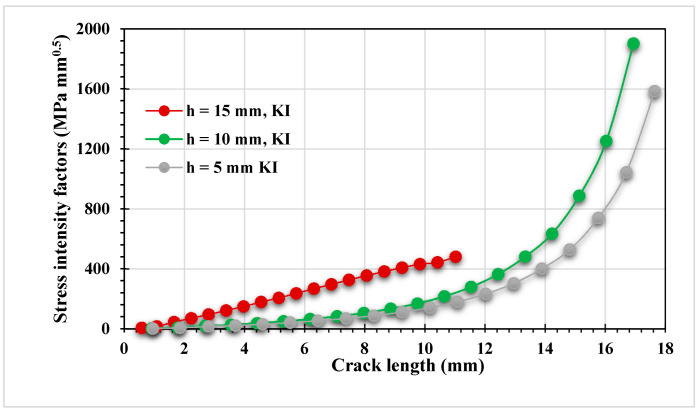
Evaluated results of *K_I_* for three pre-crack positions.

**Figure 15:**
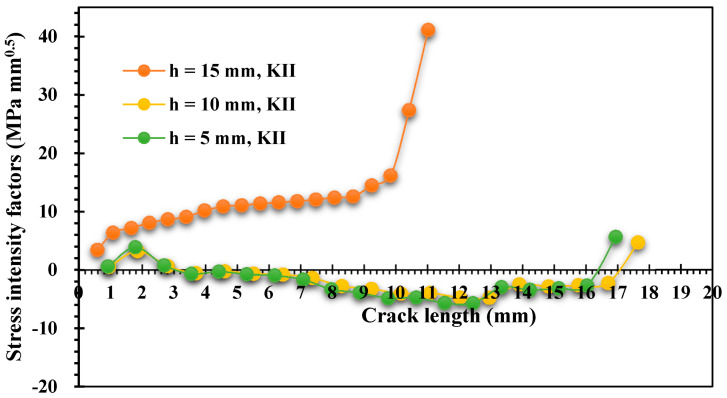
Evaluated results of *K_II_* for three pre-crack positions.

Figure 14 and Figure 15 illustrate the estimated results for *K_I_*. As shown in these figures, the case of a crack position with h = 15 has higher *K_I_* and *K_II_* results than the other two cases for the same crack length interval, demonstrating the influence of the hole on the increase of the SIFs when the crack grows towards it. As the *K_II_* values increase, the crack grows in a curvature trajectory until it falls into the hole, as shown in Figure 13a. In addition, there was a minor effect of the hole at the onset of the crack trajectory in the other two configurations with h = 10 mm and h = 5 mm, which can also be noticed in the increase of *K_II_*; nevertheless, when the *K_I_* values were increased, the crack kept growing in a straight path, and this mode completely controlled the crack propagation trajectory with a reduction in the *K_II_* values, as shown on Figure 13b,c.

## 4. Conclusions

In this study, a crack growth simulation was performed for a cracked plate with four holes and a plate with a circular hole and for an edge crack with different pre-crack positions, employing the ANSYS SMART fracture technology, which was recently introduced into the ANSYS Mechanical 19.2. The SMART crack propagation simulation was based on the Paris law and used a tetrahedral mesh for the crack fronts, which was automatically updated when the crack front changed according to the crack propagation. Due to uneven stresses at the crack tip caused by the hole, the crack grows towards the hole, as predicted. The holes act as a crack stopper, attracting a crack path that grows towards the hole, depending on the position of the hole and the initial position of the crack tip from the hole. Depending on the position of the hole, the direction of growth of the crack either deviates from the hole, which is known as the missed hole phenomenon, or it grows towards the hole and sinks into it, which is known as the sinking in hole phenomenon.

## Data Availability

The data presented in this study are available upon request from the corresponding author.
